# DCTPP1 regulates oxidative stress homeostasis via AUF1 in human villous trophoblasts

**DOI:** 10.1038/s41420-025-02666-8

**Published:** 2025-08-23

**Authors:** Yajuan Lu, Xue Wu, Lei He, Peng Pan, Anqi Zhao, Tangli Kan, Yuting Chu, Jinglin Dong, Shuangkai Xu, Xiaofang Tan, Xiaoqing Yang

**Affiliations:** 1 Institute of Reproductive Medicine, Medical School, Nantong University, Nantong, Jiangsu China; 2 Department of Obstetrics and Gynecology, Affiliated Hospital of Nantong University, Medical School of Nantong University, Nantong, China; 3 Reproductive medicine Center, Affiliated Maternity and Child Health Care Hospital of Nantong University, Medical School of Nantong University, Nantong, China

**Keywords:** Cell growth, Infertility

## Abstract

Placental trophoblast dysfunction is one of the main causes of missed abortion (MA). The expression and regulation of specific molecules play crucial roles in this complex process. Among these, human deoxycytidine triphosphate pyrophosphatase 1 (DCTPP1), a key enzyme, not only participates in nucleotide metabolism but also plays an indispensable role in maintaining genomic stability. To delve deeper into the mechanism of DCTPP1 in placental trophoblast cell function, we used an immortalized human first-trimester extravillous trophoblast cell line (HTR8/SVneo) as an experimental model for functional studies. A decrease in DCTPP1 expression leads to an increase in oxidative stress and decreased cell viability ultimately leading to apoptosis. Further analysis revealed an interaction between DCTPP1 and the AU-rich element RNA-binding protein 1 (AUF1). A decrease of AUF1 induced oxidative stress imbalance, leading to apoptosis in HTR8/SVneo cells. These findings highlight DCTPP1 as a potential biomarker and an effective drug target for the treatment or prevention of MA.

## Introduction

The placenta is an essential organ that provides oxygen, nutrients, hormones, and growth factors to the fetus throughout pregnancy, allowing material exchange between the mother and fetus [1, 2]. Its specific structural and functional abnormalities are closely related to various pregnancy complications, including insulin resistance, preeclampsia, and more severe eclampsia [3]. Placental dysfunction can affect the fetus and lead to premature birth and fetal growth issues [4]. In this process, trophoblasts play a crucial role as they have the ability to invade the decidua, which enables them to successfully obtain maternal blood supply [5, 6]. During placental development, trophoblasts specifically and persistently invade the maternal uterine tissue. Trophoblasts migrate to and invade the uterine wall, promoting remodeling of the maternal vascular system [7]. Trophoblasts form the external tissues of the embryo and play an important role in embryonic development [8].

Internal physiological processes such as cellular metabolism, external oxidative damage, and pathogen infection, can lead to the production of nonclassical nucleotides. The incorporation of nonclassical nucleotides into DNA may lead to mutagenesis and increased DNA damage, which is detrimental to the stability and integrity of the genome [9, 10]. Deoxycytidine triphosphate pyrophosphatase 1 (DCTPP1) is a recently discovered pyrophosphatase containing a bacterial MazG domain. It targets deoxycytidine triphosphate (dCTP) and hydrolyzes it into dCMP and pyrophosphate, which prevents nonclassical nucleotides from being incorporated into mitotic DNA [11, 12]. The expression of DCTPP1 is significantly high in embryonic and proliferative tissues, and its main activity site is the nucleus. At this critical position, DCTPP1 plays an important role in regulating the balance of dCTP levels in the nucleus [13]. Its function is crucial, as it can prevent excessive accumulation of dCTP within cells, which may disrupt the DNA structure and cause damage. Therefore, DCTPP1 plays an indispensable role in regulating dCTP concentration, maintaining DNA structural stability, and enhancing cellular defense against DNA damage. It can regulate the metabolism of 5-methyl-dCTP, and affect the overall methylation level of cells, thus promoting the growth and stemness of the breast cancer susceptibility gene (BRCA) cells [14]. From a broader perspective, DCTPP1 can be seen as the “steward” of the deoxyribonucleotide triphosphate (dNTP) pool. Its main biological function is to maintain genome integrity by degrading a series of nonclassical deoxycytidine analogs [15, 16]. DCTPP1 is highly expressed in various cancers and its elevated expression is associated with poor prognosis in various cancers [11]. Research on the coding gene *DCTPP1*, is currently focused on exploring its complex association with tumor cells. Scientists are increasingly interested in DCTPP1 because it exhibits unique expression patterns and functional properties in various tumor cells [17].

Heterogeneous ribonucleoprotein D (HNRNPD), also known as AU binding factor 1 (AUF1), is one of the well-known RNA-binding proteins (RBPs), and the first translation and turnover (TTR)-RBP to be isolated and proven to control mRNA stability [18]. The recognition and binding of AUF1 to its targeted mRNA mainly rely on the AU-rich elements located in the 3’-untranslated region of mRNA. Although the specific mechanism is not completely clear, AUF1 promotes the degradation of the target mRNA and enhances its stability and translation efficiency [19, 20]. In addition to regulating mRNA stability, AUF1 also plays multiple roles in the nucleus, including telomere maintenance, transcriptional activation, and selective splicing [21]. The target genes of AUF1 are widely distributed in many physiological processes such as cell proliferation, apoptosis, aging, and metastasis, which have crucial impacts on growth, development, and disease occurrence in organisms [22, 23]. For instance, by up-regulating NRF2 and down-regulating ATF3, AUF1 antagonizes ferroptosis in alveolar epithelial cells in vitro [24]. By down-regulating ZBTB2 and subsequently TRIM58, AUF1 can contribute to the aggressive behavior of thyroid cancer cells, including increased proliferation and invasion [25]. Protein phosphorylation and other post-translational modifications regulate AUF1 function by affecting RNA folding, cellular localization, RNA binding, protein stability, and protein interactions [21].

Based on the discovery of the role of DCTPP1 in tumors and its decreased expression in trophoblast cells of patients with missed delivery, we speculated that DCTPP1 may also have an impact on trophoblast cells. Therefore, this study aimed to determine the role of DCTPP1 in HTR8/SVneo cells during pregnancy. HTR8/SVneo is a human villus trophoblastic cell line commonly used in the study of gestational diseases and the biological functions of the placenta. The potential involvement of DCTPP1 in human trophoblasts, particularly its interaction with the AUF1 protein. This is of great significance for understanding embryo implantation, placental formation and regulation, as well as the prevention and treatment of pregnancy related placental diseases.

## Results

### DCTPP1 knockdown induced oxidative stress imbalance in HTR8/SVneo cells

**Fig. 1:**
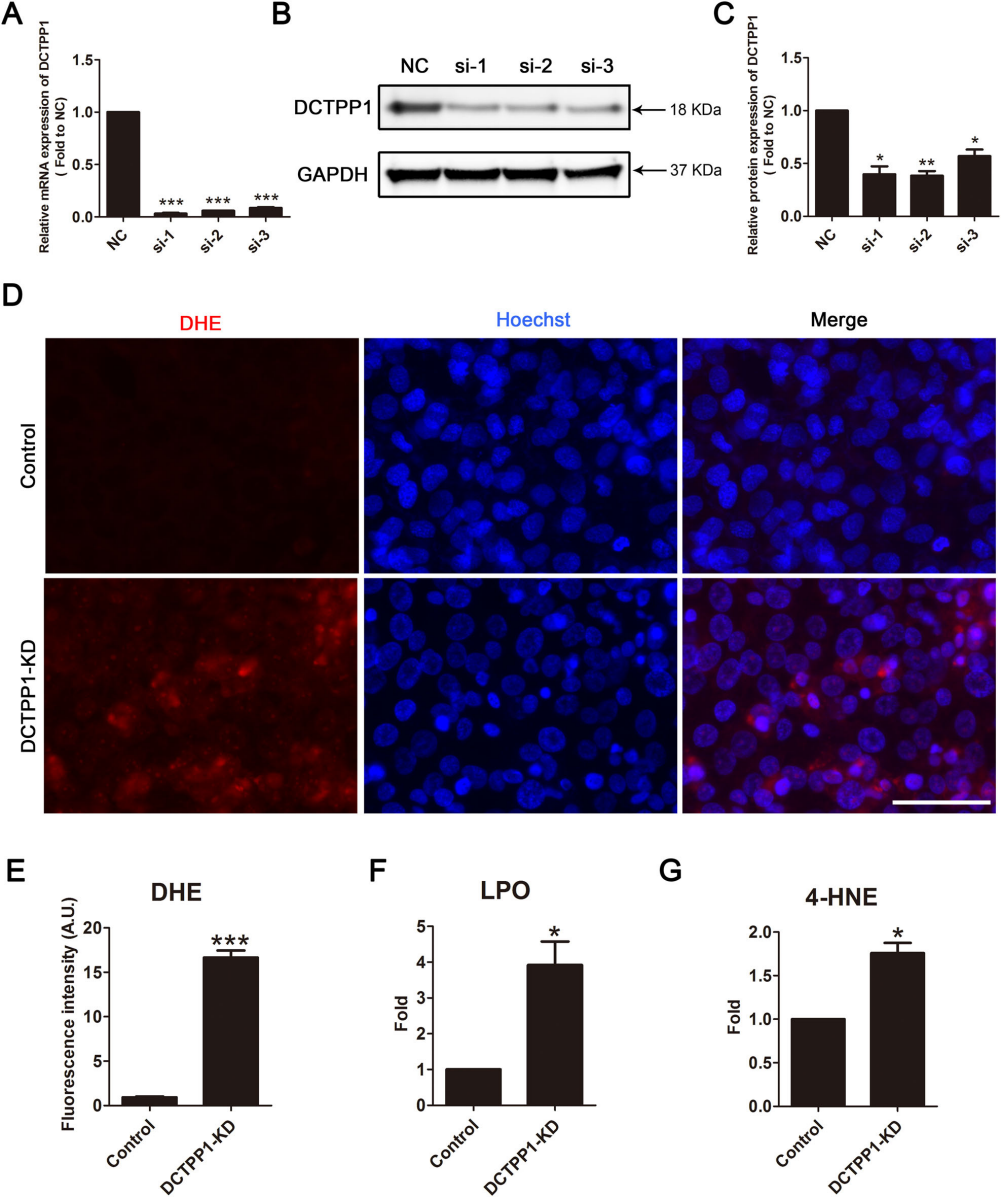
ROS generation was increased in HTR8/SVneo cells by DCTPP1 knocking down. **A** qRT-PCR was used to detect the efficiency of three siRNA-mediated knockdown of DCTPP1. **B** DCTPP1 with different siRNA were measured using western blotting. **C** Relative protein expression of DCTPP1. **D** ROS was measured using a DHE assay and immunofluorescence images were observed with a scale of 50 μm. **E** Schematic of fluorescence intensity in DCTPP1-KD and control groups. **F** Relative LPO contents of the DCTPP1-KD group compared with that of the control. **G** Relative 4-HNE concentration of the DCTPP1-KD group when compared with that of the control. *P < 0.05, **P < 0.01, and ***P < 0.001.

To identify the specific role of DCTPP1 in placentation, we examined how DCTPP1 affects the function of HTR8/SVneo cells. As shown in Fig. 1A–C, we used siRNA to knock down the expression levels of DCTPP1 and identified siRNA-1 as the most efficient siRNA. We used this siRNA to knock down DCTPP1 and detected ROS levels using immunofluorescence staining with DHE. The fluorescence intensity of DHE in the knockdown group was significantly higher than that in the control group (Fig. 1D, E). Additionally, the increased absorbance values of LPO in the knockdown group also proved that the decrease of DCTPP1 would cause the increase of oxidative stress (Fig. 1F). The 4-HNE index was also determined in order to confirm lipid peroxidation in the DCTPP1 knockdown group (Fig. 1G).

### Knockdown of DCTPP1 impaired cell proliferation and induced cell death in HTR8/SVneo cells

**Fig. 2:**
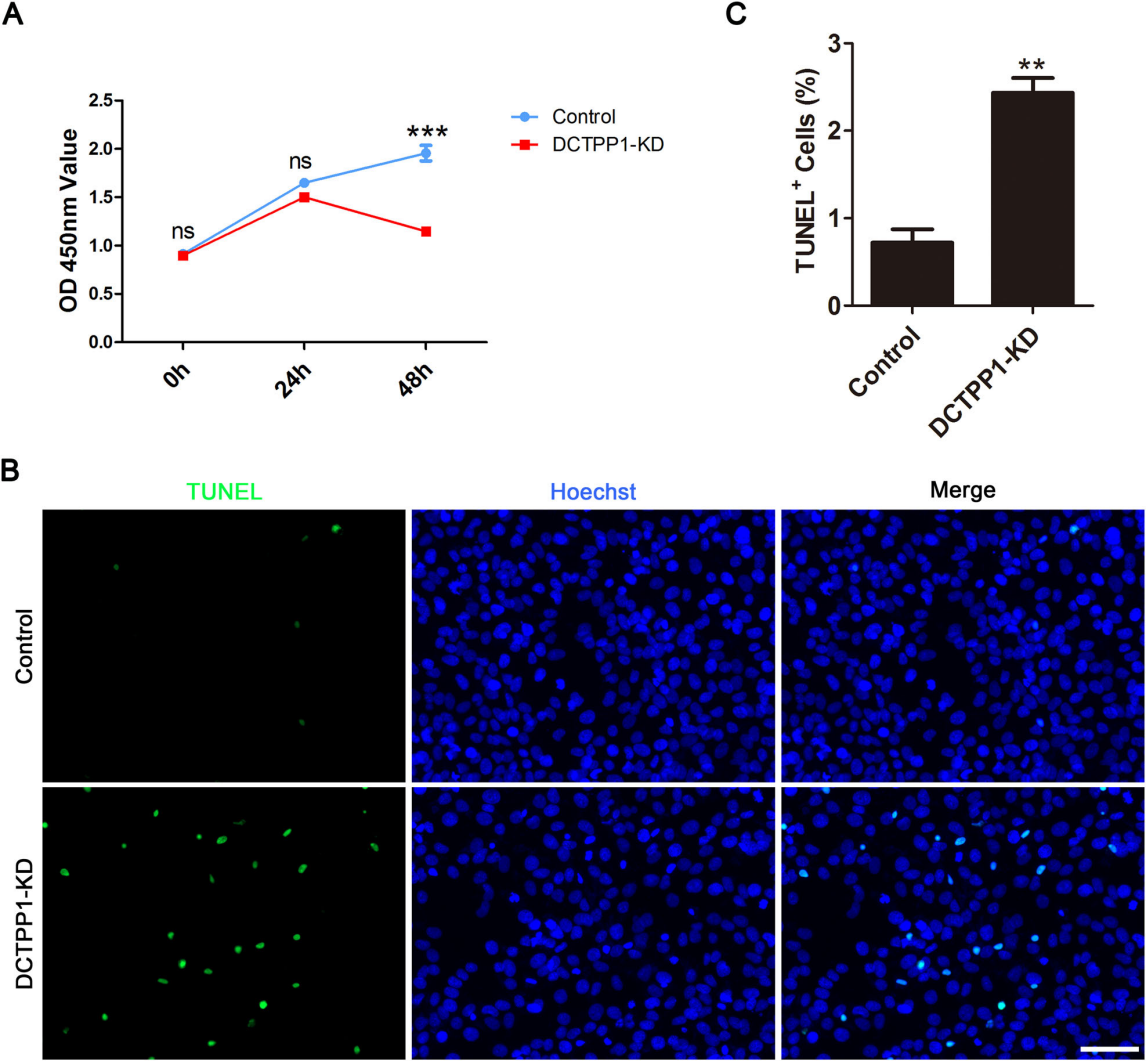
DCTPP1-KD led to the inhibition of cell proliferation and cell death. **A** CCK-8 assay for HTR8/SVneo cell in control and DCTPP1-KD groups. **B** TUNEL staining for HTR8/SVneo cell in control and DCTPP1-KD groups. DNA was stained with Hoechst; scale bar = 100 μm. **C** The percentage of TUNEL-positive cells. ns: no significance, **P < 0.01, and ***P < 0.001.

To further evaluate the function of DCTPP1 in HTR8/SVneo cells, the CCK-8 assay and TUNEL staining were used to detect whether DCTPP1 affected cell growth and apoptosis. The CCK-8 assay demonstrated that DCTPP1 knockdown dramatically affected cell growth after 48 h compared with that of the control (Fig. 2A). Consistent with the cell growth ability, we also showed that after 48 h of knockdown, the ratio of TUNEL-positive cells in the knockdown group increased significantly compared to that in the control group (Fig. 2B, C).

### Identifying the target pathway of DCTPP1 in HTR8/SVneo cells using transcriptome analysis

**Fig. 3:**
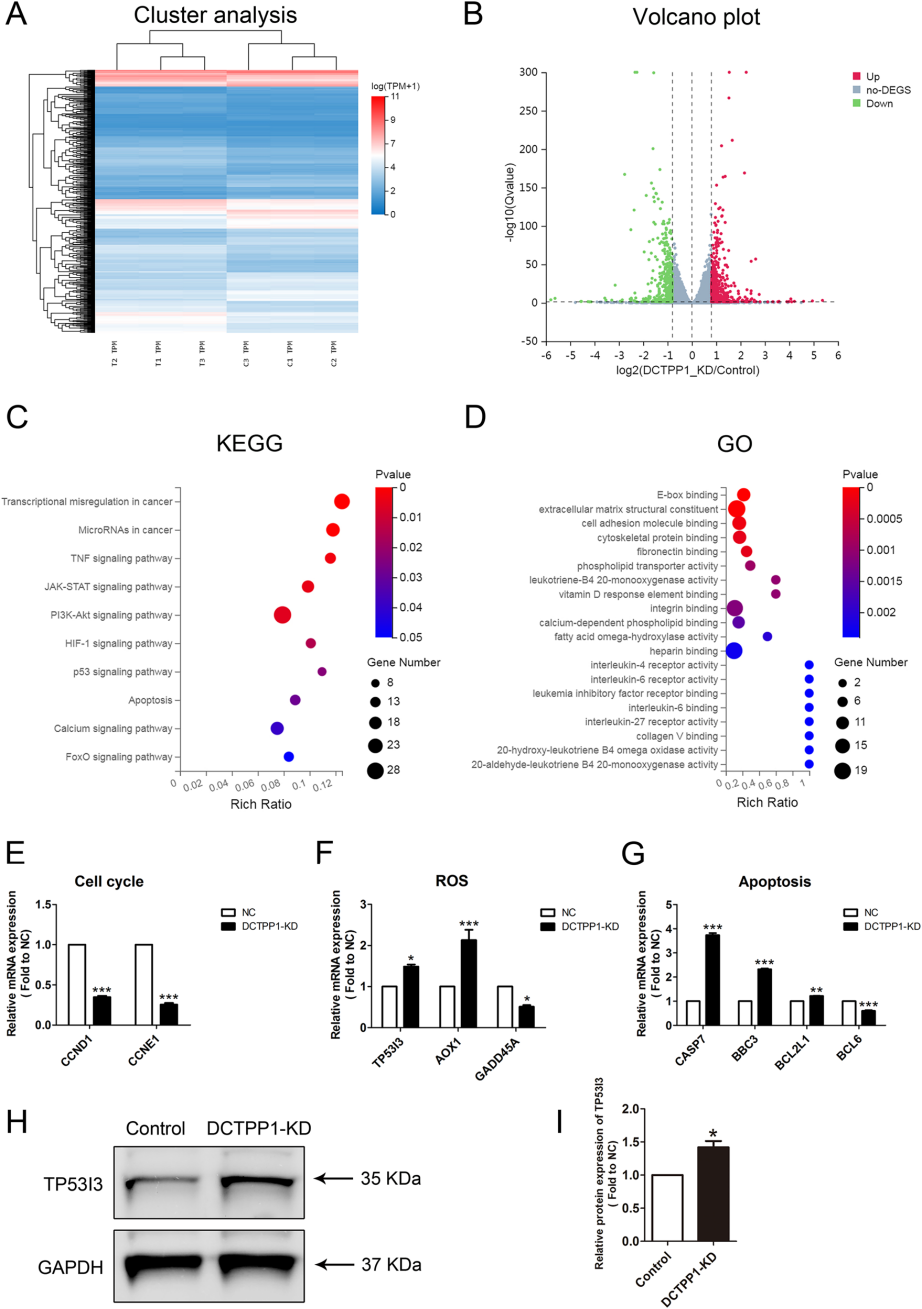
Transcriptome sequencing analyzed the effect of DCTPP1-KD in HTR8/SVneo cells. **A** Cluster analysis plot of differentially expressed genes (DEGs) after DCTPP1 knockdown. C1, C2, C3 and T1, T2, T3 indicate independent data replicates for each group. **B** Volcanic map analysis of DEGs in DCTPP1-KD and control groups. Differential genes are color coded. **C** KEGG enrichment analysis enriched DEGs factors after DCTPP1 knockdown. **D** The GO analysis showing the function linked to the DEGs. **E** Validation of RNA-seq data related to cell cycle between DCTPP1-KD and control groups using qRT-PCR. **F** Validation of RNA-seq data related to ROS in DCTPP1-KD and control groups using qRT-PCR. **G** Validation of RNA-seq data related to apoptosis in DCTPP1-KD and control groups using qRT-PCR. **H** Representative western blot images showing TP53I3 expression in DCTPP1-KD cells when compared to controls, with GAPDH serving as the loading control. **I** Quantitative analysis of TP53I3 protein expression. *P < 0.05, **P < 0.01, and ***P < 0.001.

To elucidate the underlying mechanisms by which DCTPP1 influences the function of HTR8/SVneo cells, RNA sequencing was used to conduct a comprehensive analysis of the transcriptomes of DCTPP1-knockdown and control cells (three repetitions in each group). Heatmaps and volcano plots revealed 889 differentially expressed genes (DEGs) were successfully identified between the knockdown and control group (Fig. 3A, B, and Table S1). Among these 889 DEGs, over 500 were upregulated and over 300 were downregulated (Fig. 3B). Notably, further KEGG analysis revealed that these DEGs were significantly enriched in apoptosis and the FOXO signaling pathway (Fig. 3C). Additionally, GO analysis provided detailed information on the DEGs involved in oxidative stress-related molecular functions (Fig. 3D). We validated these results by assessing the expression levels of selected transcripts linked to cell cycle (CCND1 and CCNE1), ROS signaling (TP53I3, AOX1 and GADD45A), and apoptosis (CASP7, BBC3, BCL2L1 and BCL6) using qRT-PCR (Fig. 3E–G). Notably, the reliability of the detected ROS-associated TP53I3 pathway was confirmed using western blotting (Fig. 3H, I). Overall, these results indicate that the knockdown of DCTPP1 significantly affects the expression of multiple genes at the transcriptional level, particularly those related to oxidative stress, providing compelling evidence for the mechanisms by which DCTPP1 influences HTR8/SVneo cells.

### DCTPP1 physically interacted with AUF1

**Fig. 4:**
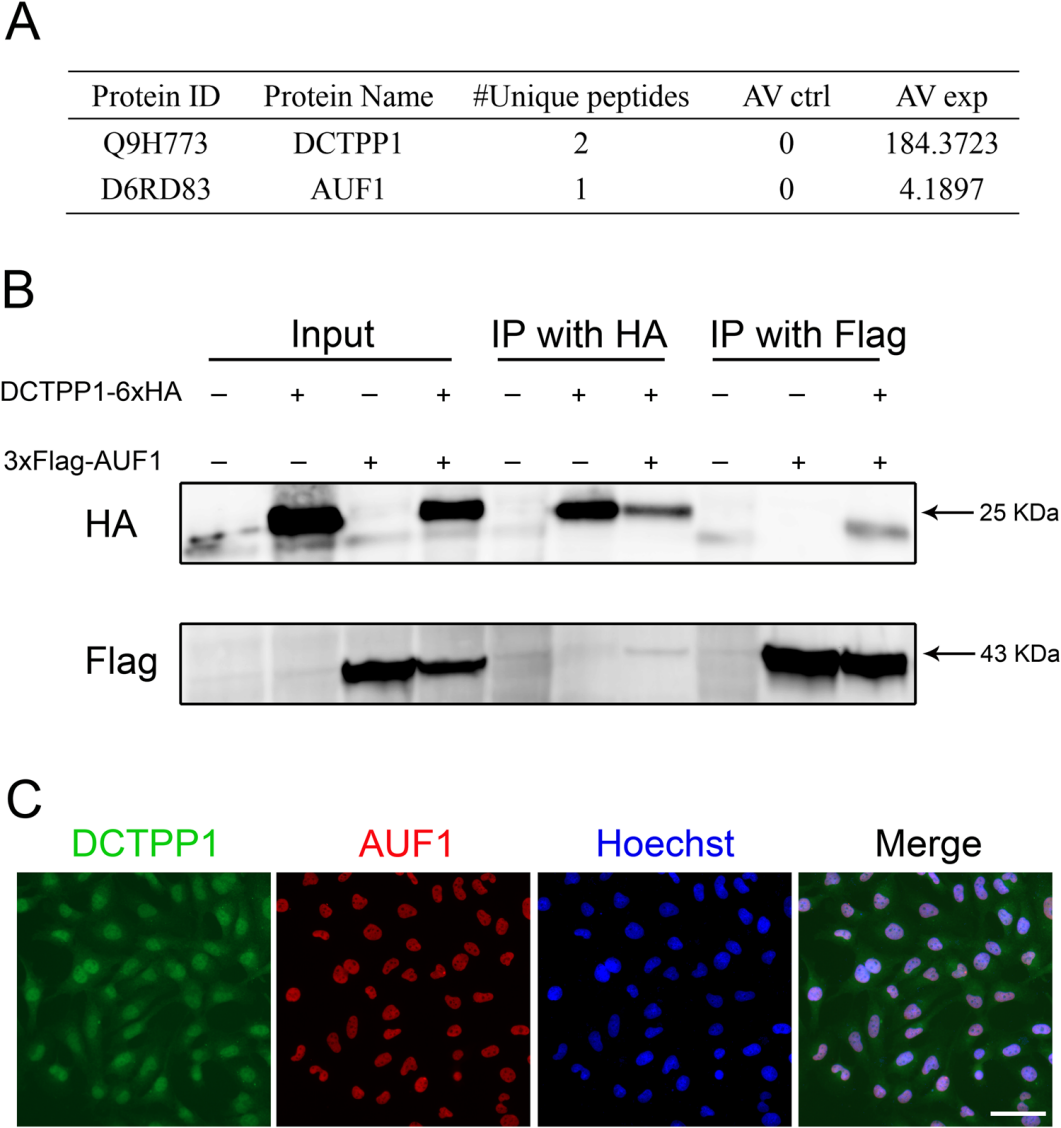
The interaction between DCTPP1 and AUF1. **A** Identification of binding partner of DCTPPl by immunoprecipitation and MS. **B** Co-IP was performed to determine the interaction between DCTPPl and AUF1. **C** Representative immunofluorescence images showing co-localization of DCTPP1 and AUF1 in cells. scale bar = 50 μm.

To gain insight into the molecular function of DCTPP1 in oxidative stress and apoptosis, we used immunoprecipitation and mass spectrometry (MS) to identify its potential downstream effectors. From the MS results, we found that AUF1, play a role in the elimination of oxidized RNA [26] and binds to DCTPP1 (Fig. 4A). We verified the interaction between DCTPP1 and AUF1 using co-IP experiments with an overexpression vector in HTR8/SVneo cells. IP using either HA or Flag-tagged magnetic beads precipitated the corresponding tagged target protein and its binding partner with another tag (Fig. 4B). Subsequently, we confirmed the co-localization of DCTPP1 and AUF1 in HTR8/SVneo cells through immunofluorescence assays with their respective antibodies (Fig. 4C). These results demonstrate a physical interaction between DCTPP1 and AUF1 in HTR8/SVneo cells.

### AUF1 knockdown induced increased ROS and apoptosis in HTR8/SVneo cells

**Fig. 5:**
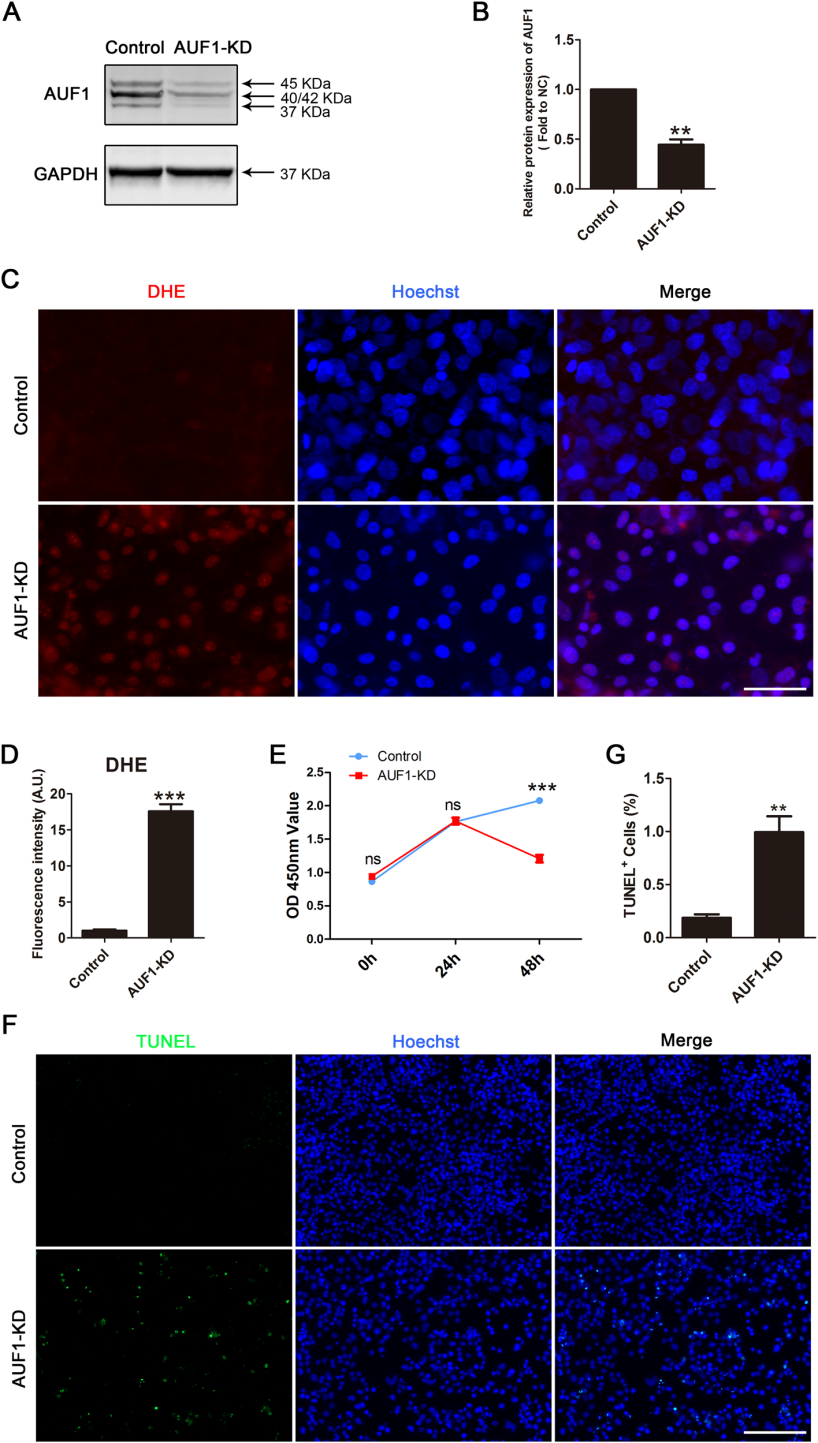
Silencing *AUF1* gene caused excessive oxidative stress and cell apoptosis in HTR8/SVneo cells. **A** The band of AUF1 knockdown efficiency was measured using western blotting. **B** Relative protein expression of AUF1. **C** ROS production was determined using the DHE assay and immunofluorescence images were shown, scale bar = 50 μm. **D** Quantification of DHE fluorescence intensity in control and AUF1-KD groups. **E** CCK-8 assay for HTR8/SVneo cell in control and AUF1-KD groups. **F** TUNEL staining for HTR8/SVneo cells in control and AUF1-KD groups. DNA was stained with Hoechst, scale bar = 200 μm. **G** The percentage of TUNEL-positive cells. ns: no significance, **P < 0.01, and ***P < 0.001.

To confirm the role of AUF1 in oxidative stress in HTR8/SVneo cells, we performed knockdown experiments. First, the knockdown efficiency of the AUF1 protein was achieved using a specific siRNA (Fig. 5A, B). The immunofluorescence pattern and intensity calculation of DHE confirmed that the degree of oxidative stress in cells increased after AUF1 knockdown (Fig. 5C, D). Due to the influence of elevated ROS, cell proliferation was significantly reduced after 48 h in the AUF1–knockdown group when compared with that in the control group (Fig. 5E). TUNEL staining also showed that the apoptosis rate in the AUF1–knockdown group was significantly higher than that in the control group (Fig. 5F, G). These results suggested that AUF1 plays a role in regulating oxidative stress levels in HTR8/SVneo cells.

### Identification of the AUF1-mediated transcriptional regulatory network in HTR8/SVneo cells

**Fig. 6:**
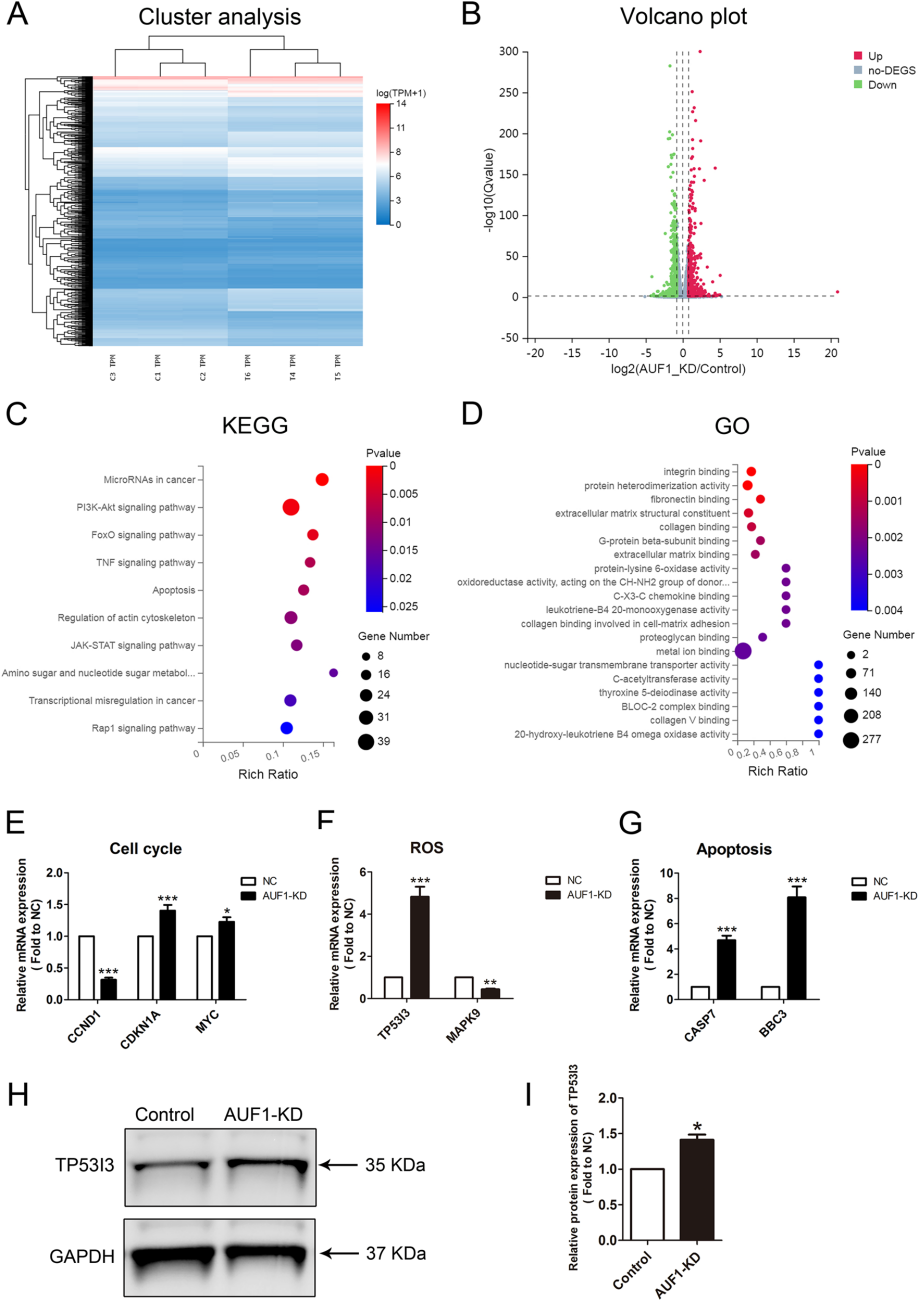
Transcriptome sequencing analyzed the effect of AUF1-KD in HTR8/SVneo cells. **A** Cluster analysis plot of DEGs after AUF1 knockdown. C1, C2, C3 and T1, T2, T3 indicate independent data replicates for each group. **B** Volcanic map analysis of DEGs in AUF1-KD and control groups. Differential genes are color coded. **C** KEGG analysis enriched DEGs after AUF1 knockdown. **D** The GO analysis showing the function linked to the DEGs. **E** Validation of RNA-seq data related to the cell cycle between the AUF1-KD and control groups using qRT-PCR. **F** Validation of RNA-seq data related to ROS between AUF1-KD and control groups using qRT-PCR. **G** Validation of RNA-seq data related to apoptosis in AUF1-KD and control groups using qRT-PCR. **H** The western blot images of TP53I3 and GAPDH in AUF1-KD and control cells. **I** Bar graph showing relative TP53I3 protein expression (normalized to GAPDH). *P < 0.05, **P < 0.01, and ***P < 0.001.

To investigate how AUF1 affects the transcriptional regulatory network in HTR8/SVneo cells, we performed RNA-seq on cells in AUF1–knockdown and control cells. Cluster analysis and volcano plots revealed decreased expression of 601 genes and increased expression of 586 genes (Fig. 6A, B and Table S2). Similar to DCTPP1 knockdown, KEGG analysis showed that the DEGs were significantly enriched in the apoptosis and FOXO signaling pathways (Fig. 6C). In addition, GO analysis revealed that DEGs were involved in the molecular function of oxidative stress (Fig. 6D). Regarding the molecular mechanisms, AUF1 and DCTPP1 were mostly identical. The three biological events of cell cycle (CCND1, CDKN1A and MYC), ROS production (TP53I3 and MAPK9), and apoptosis (CASP7 and BBC3) were also validated using qRT-PCR with related genes (Fig. 6E–G). Moreover, these genes partially overlap with DCTPP1 pathway-related genes. We further verified the reliability of the observed ROS-linked TP53I3 pathway, which functions consistently with DCTPP1 through western blotting (Fig. 6H, I).

## Discussion

DCTPP1 significantly contributes to cellular nucleotide metabolism by ensuring the stability of the dNTP pool and effectively preventing the invasion of nuclear DNA by oxidative damage [27]. The high evolutionary conservation of DCTPP1 across mammals underscores its biological importance, and its elevated expression in highly proliferative cells supports DNA replication and genome maintenance [28]. In this study, we found that compared with healthy individuals, patients with MA had significantly lower levels of RNA expression of DCTPP1 in placental trophoblast cells. However, the specific mechanism of DCTPP1 in embryonic trophoblast cells is still unclear. Therefore, a series of carefully designed in vitro experiments was conducted in this study to strongly confirm the important role of DCTPP1 in regulating oxidative stress response in HTR8/SVneo cells.

To investigate the role of DCTPP1 in HTR8/SVneo cells, we adopted a targeted strategy to effectively silencing the expression of DCTPP1 using specific DCTPP1 siRNA. After a series of rigorous experimental studies, we made the following important findings: The experimental results from DHE staining, LPO, and 4-HNE detection consistently demonstrated that DCTPP1 knockdown significantly increased intracellular ROS levels, while the growth rate of trophoblast cells slowed down accordingly. These changes ultimately promoted apoptosis. Based on these observations, we speculated that DCTPP1 likely plays a key role as a redox protective protein in trophoblast cells, which is crucial for maintaining normal placental function.

ROS are a highly unstable class of free radical molecules that play crucial roles in the body as signaling molecules that regulate various cellular processes, including cell proliferation, differentiation, migration, inflammatory response, and stress adaptation [29]. However, when the level of ROS abnormally increases, the redox balance of the cells tilts towards an oxidative state. At this point, ROS will act like a fierce “invader”, attacking important biomolecules, such as DNA, lipids, and proteins, and may cause damage to organelles. This series of chain reactions ultimately lead to the development of various diseases. Therefore, it is important to develop an efficient antioxidant defense system that strictly controls ROS production [30]. During the critical period of embryo implantation and early placental formation, trophoblasts may undergo complex physiological processes, such as ischemia reperfusion and hypoxic reoxygenation. Simultaneously, during normal pregnancy, trophoblasts must withstand certain physiological pressures to maintain normal embryo development [31]. Wang et al. reported cisplatin-induced DCTPP1 upregulation via ROS in ovarian cancer cells. They found that, DCTPP1 knockdown increased oxidative stress, DNA damage, and apoptosis, which enhanced cisplatin efficacy [27]. The study further showed that DCTPP1 depletion attenuated redox gene expression and PI3K/Akt signaling, while reversing chemoresistance in SK-OV-3/DDP cells through altered antioxidant responses and ROS accumulation [32].

In our in-depth research, we unexpectedly discovered an interesting interaction; there was a close relationship between the activity of DCTPP1 and the AUF1 protein in HTR8/SVneo cells. AUF1 not only participates in the conversion of mRNA and regulates key links in gene expression, but also plays an important role in translation regulation, affecting the rate of protein synthesis [33–36]. After siRNA knockdown of AUF1, HTR8/SVneo cells showed the same elevated oxidative stress, inhibition of cell proliferation, and apoptosis as those with DCTPP1 knockdown. Transcriptome sequencing revealed that AUF1 and DCTPP1 affected the same molecular pathways involved in cellular oxidative stress, cell cycle and apoptosis. Especially in the ROS pathway, P53 inducible gene 3 (PIG3 or TP53I3) was identified in an analysis of genes induced by p53 before the onset of apoptosis and induced by oxidative stress [37]. The upregulation of TP53I3 in cells with knockdown of the two genes suggests an increase in ROS levels.

Conclusive evidence suggests that AUF1 plays an important role in the specific degradation process of oxidized RNA in living organisms [38]. Extensive studies have been conducted to elucidate this mechanism. Recent studies have revealed two key proteins that bind tightly to oxidized RNA and play important roles. One of these is the AUF1 protein, which has a unique recognition ability and participates in the selective degradation of mRNA in cells under oxidative stress [26]. Another binding protein, PCBP1, may participate in the process of inducing cell apoptosis by forming stable complexes with RNA containing 8-oxoguanine [39]. In this study, DCTPP1 was found to regulate the level of oxidative stress in trophoblasts through AUF1. DCTPP1 may modulate AUF1’s RNA-binding activity, altering the stability of the transcripts involved in oxidative stress.

In summary, we found that DCTPP1 inhibited excessive oxidative stress in HTR8/SVneo cells, thereby promoting cell proliferation and reducing apoptosis. In addition, we identified the interaction molecule, AUF1, with DCTPP1 in HTR8/SVneo cells. These results provide novel insights into DCTPP1 detection in embryonic trophoblasts from the placental function of MA. The apparent dual roles of DCTPP1 pro-tumor in some contexts yet protective in trophoblasts may indeed reflect tissue-specific functions. The differential expression patterns, interacting partners, and signaling pathways across cell types may explain these opposing effects.

## Materials and methods

### Cell culture and transient transfection of siRNA

The HTR8/SVneo cell line was purchased from KeyGEN BioTECH (Cat# KG557, Nanjing, China) and cultured in DMEM/F12 medium containing 10% fetal bovine serum at 37 °C with 5% CO_2_. Cells were seeded in multi-well plates and randomly assigned to treatment or control groups using a computer-generated randomization sequence. Transfection conditions were allocated to wells based on this randomization scheme to minimize positional bias. All the siRNAs used in this study were purchased from GenePharma Co. (Suzhou, China). The target sequences of the DCTPP1 siRNA were as follows: si-DCTPP1-1: CCUCCAUGCUGAGUUUGCUTT; si-DCTPP1-2: GCCCUUCAAGAGGAGCUUATT; and si-DCTPP1-3: GCUCUUCCCGCAAGUAUACTT. The target sequence of the AUF1 siRNA was AGACUGCACUCUGAAGUUATT. When the cell density reached 70%, Lipofectamine 3000 reagent (Invitrogen, USA) was used to transfect siRNAs in Opti-MEM Reduced Serum Medium (Gibico, USA) according to the standard protocol. The transfection medium was replaced with the complete medium 6 h after transfection. Cells were collected 24 and 48 h later for qRT-PCR and western blotting, respectively.

### Quantitative real-time PCR (qRT-PCR)

TRIzol reagent (Invitrogen) was used to extract total RNA from the samples. Subsequently, 200 ng of RNA was reverse transcribed using the PrimeScript RT Master Mix (Cat# RR036A, Takara, Kyoto, Japan). When setting up the PCR reaction mixture, each reaction tube was loaded with 10 μL of TB Green Premix (Takara), 7.8 μL of water, 1 μL of cDNA sample, and 1.2 μL of gene-specific primers (Supplemented materials). Next, we used Light Cycler 96 real-time quantitative PCR system (Roche, Switzerland) to measure the expression levels of the target genes. To ensure the accuracy of the experimental results, at least three independent experiments were conducted, and the relative gene expression of actin was calculated using the 2^−△△Ct^ method.

### Western blotting and analysis

Cells from each group were lysed in cell lysis buffer, placed on ice, and the total protein was successfully extracted. Subsequently, the concentrations of the extracted proteins were determined using a BCA kit. Then, 30 μg of protein was separated using 10% SDS-PAGE gel and transferred to a PVDF membrane. During the blocking step, the PVDF membrane was treated with blocking buffer provided by Beyotime Biotechnology (China). After blocking, the membranes were incubated overnight at 4 °C with primary antibodies against DCTPP1 (dilution ratio 1:1000; Abcam, cat No. ab224051), AUF1 (dilution ratio 1:1000; Proteintech, cat No. 12770-1-AP), TP53I3 (dilution ratio 1:1000; Proteintech, cat No. 14828-1-AP), and GAPDH (dilution ratio 1:3000; Proteintech, cat No. 60004-1-Ig). The next day, after washing, the secondary antibody was incubated for 1 h at 25 °C. Finally, the protein bands were visualized using the Amersham Typhoon Odyssey infrared imaging system.

Western blot bands were quantified using Image J software by converting images to 8-bit grayscale and then applying background subtraction. Target protein bands were selected using rectangular tool and their integrated densities (IntDen) were measured. Data were normalized to the loading control (GAPDH) and expressed as fold-changes relative to the controls. Statistical analyses were performed on exported data.

### Reactive oxygen species (ROS) assay

Dihydroethidium (DHE, S0063, Beyotime Biotechnology) was used to detect ROS generation based on the fluorescence intensity. After ingestion by living cells, DHE is dehydrogenated by superoxide anions to produce ethidium. Ethidium binds to RNA or DNA to produce red fluorescence. Cells from various groups were plated in 24-well plates with round coverslips and incubated with DHE probes in a completely dark environment for 30 min. Subsequently, the DHE probe was removed, and the cells were carefully washed twice with phosphate-buffered saline (PBS). Cells were fixed with 4% paraformaldehyde for 30 min and stained with Hoechst dye for 10 min. Finally, the processed cells were carefully placed on glass slides, observed, and analyzed in detail under a fluorescence laser scanning microscope (Axio Imager M2, Zeiss, Germany).

### Oxidative stress assay

A lipid peroxidation (LPO) assay kit (BC5245, Solarbio) was used to assess oxidative stress. Under acidic conditions, the samples were heated, causing the LPO to decompose and produce malondialdehyde (MDA). Subsequently, MDA underwent condensation with thiobarbituric acid, forming a brownish-red substance known as trimethyl ester (with the specific chemical formula of 3,5,5-trimethyloxazole-2,4-dione). The trimethyl ester exhibited maximum light absorption at a wavelength of 532 nm. Following the manufacturer’s instructions, the relative LPO content in the samples was calculated by measuring absorbance, thereby indirectly reflecting the degree of oxidative stress.

### 4-HNE Detection

The concentration of 4-hydroxynonenal (4-HNE) in HTR8 cells was quantified using a 4-HNE ELISA kit (Elabscience, Cat# E-EL-0128). Briefly, the cell samples were incubated with the kit’s dye solution at 37 °C in the dark for 20 min. The absorbance was measured at 450 nm using a microplate reader (BioTek, USA). Absolute 4-HNE concentrations were calculated based on a standard curve and the data were normalized to those of the control group (set as 1) to express the results as the relative ratio of 4-HNE levels in the treated vs. control groups. All of the experiments were independently repeated thrice.

### Cell counting kit-8 (CCK-8) assay

Cells were seeded in the 96-well plate at 5000 cells/100 μL/well and transfected with DCTPP1 or AUF1 siRNA, respectively. CCK-8 reagent (Beyotime Biotechnology) was added to each well, and the cells were incubated for another 2 h at 0, 24 and 48 h after transfection. The OD450 nm value was measured using a microplate reader.

### TUNEL assay

A one-step TUNEL apoptosis assay kit (C1086, Beyotime Biotechnology) was used to detect the apoptosis rates of cells after *DCTPP1* and *AUF1* gene knockdown. Fluorescein-labeled dUTP can be added to exposed 3’-OH exposed to genomic DNA breaks catalyzed by terminal deoxynucleotidyl transferase (TdT). The reaction solution, prepared before the assay, was a mixture of buffer, dUTP, and TdT at a ratio of 50:5:1. The reaction solution was then incubated with cells in a humidified environment at 37° C for 2 h. After incubation, the cells were washed thrice with PBS and stained with Hoechst dye for 10 min. Finally, the processed cells were observed under a fluorescence laser scanning microscope to assess their apoptotic status.

### Mass spectrometry analysis

The protein samples were reduced and alkylated prior to in-gel tryptic digestion. The resulting peptides were separated by nanoLC and analyzed using an Orbitrap Fusion Lumos mass spectrometer in the data-dependent acquisition mode. Full-scan MS1 spectra were acquired at a resolution of 60,000 (mass range: 350–1500 *m*/*z*), followed by MS/MS fragmentation via higher-energy collisional dissociation (HCD) at 15,000 resolution. Raw data were processed using MaxQuant software (version 1.6.5.0) for peptide identification and quantification.

### Plasmid construction and transient transfection

*DCTPP1* cDNA was sub-cloned into pcDNA3.1-6×HA vector and *AUF1* cDNA was sub-cloned into the p3×FLAG-myc-CMV-24 expression vector. When the cell density reached 70%, Lipofectamine 3000 reagent (Invitrogen) was used to transfect vectors in Opti-MEM Reduced Serum Medium (Gibco) according to the standard protocol. The transfection medium was replaced with the complete medium 6 h after transfection. The cells were collected 24 h later for immunoprecipitation (IP) and western blotting.

### Co-IP

At 24 h post-transfection, the cells were lysed using mild RIPA lysis buffer (Beyotime; protease inhibitors were added before use). Subsequently, cell lysates were incubated in an ice bath for 20 min to ensure complete cell lysis. Cell debris was removed using high-speed centrifugation, and the supernatant was collected. Next, the supernatant was incubated overnight at 4 °C with magnetic beads specifically recognizing HA or FLAG tags (MedChemExpress), allowing the target proteins to fully bind to the beads. After incubation, the beads were washed thrice with wash buffer to remove unbound impurities. After washing, proteins bound to the beads were eluted with SDS buffer. Finally, the diluted protein samples were analyzed using immunoblotting to assess the expression levels of the target proteins.

### Immunofluorescence Staining

Coverslips were pretreated with poly-L-lysine for 30 min at room temperature (RT), followed by three washes and air-drying prior to cell seeding. The digested cells were cultured on the prepared coverslips in 24-well plates until adherence. Following fixation with 4% paraformaldehyde (30 min at RT) and permeabilization with 0.5% Triton X-100 (20 min at RT), samples were blocked with 1% BSA (60 min at RT). Cells were then incubated with DCTPP1 antibody (same as western blot) diluted in blocking buffer overnight at 4 °C, washed with PBST, and subsequently treated with Goat anti-Rabbit IgG (H + L) Cross-Adsorbed Secondary Antibody, Alexa Fluor™ 488 (Thermo Fisher, cat No.11008) for 1 h at RT (light-protected). After washing, AUF1 antibody (Proteintech, cat No.68236-1-Ig) staining using the same protocol and incubation with Donkey anti-Mouse IgG (H + L) Highly Cross-Adsorbed Secondary Antibody, Alexa Fluor™ 555 (Thermo Fisher, cat No.A-31570) were performed. Nuclear counterstaining was performed using Hoechst (10 min at RT) before fluorescence imaging acquisition using a microscope system.

### Transcriptome sequencing and analysis

The TRIzol reagent was used to extract total RNA from HTR8/SVneo cells in both the control and treatment groups. Subsequently, mRNA was enriched from these total RNA samples, and transcriptome libraries were constructed based on this mRNA. These libraries were then sequenced using the DNBSEQ high-throughput sequencing platform, and the obtained data were subjected to in-depth bioinformatics analysis. This series of RNA studies was performed by BGI Genomics Co., Ltd. During bioinformatics analysis, the expression levels of each gene were first calculated using the RSEM software (version v1.3.1).

Based on the differences in gene expression among different samples, a visual heatmap was generated using the pheatmap software (version v1.0.12). For differential expression analysis, the DESeq2 software was used, with a Q-value ≤ 0.05 set as the significance threshold. In the resulting volcano plot, differentially expressed genes (DEGs) were color coded: genes with a log2 (fold change) > 0.8 and Padj < 0.05 were highlighted in red, indicating upregulation; genes with a log2 (fold change) < −0.8 and Padj < 0.05 were marked in green, indicating downregulation; and gray represented genes with no significant difference. To gain a deeper understanding of the impact of these DEGs on cellular phenotypes, the Phyper method, based on a hypergeometric test, was adopted to perform Gene Ontology (GO) and Kyoto Encyclopedia of Genes and Genomes (KEGG) enrichment analyses of the annotated DEGs. In the enrichment analysis, strict thresholds (*Q*-value ≤ 0.05) were applied to correct the significance levels of terms and pathways, ensuring the accuracy and reliability of the analysis results.

### Statistical analysis

Statistical analyses were conducted using GraphPad PRISM version 5 using Student’s *t*-test or one-way analysis of variance (ANOVA) followed by Dunnett’s test for multiple comparisons (Compare all columns versus the control column). The student’s *t*-test was used when only two data sets were compared. All experiments were repeated at least three times and data were expressed as mean ± SEM. Data were considered statistically significant when the *p*-value was less than 0.05.

## Supplementary information

Primer sequences

Original western blots

Table S1. DEGs of DCTPP1-KD in HTR8/SVneo cells.

Table S2. DEGs of AUF1-KD in HTR8/SVneo cells.

## Data Availability

The raw RNA sequence data reported in this paper have been deposited in the Genome Sequence Archive (Genomics, Proteomics & Bioinformatics 2021) in National Genomics Data Center (Nucleic Acids Res 2022), China National Center for Bioinformation / Beijing Institute of Genomics, Chinese Academy of Sciences (GSA-Human: HRA011864) that are publicly accessible at https://ngdc.cncb.ac.cn/gsa-human. The mass spectrometry proteomics data have been deposited to the Proteome X change Consortium via the PRIDE partner repository with the dataset identifier PXD065017.
