# Supplementary material for: DCTPP1 regulates oxidative stress homeostasis via AUF1 in human villous trophoblasts
Source: Cell Death Discov. 2025 Aug 23;11:400. doi: 10.1038/s41420-025-02666-8 (PMC12375105; doi:10.1038/s41420-025-02666-8)
Supplement: Supplementary file 1 — Primer sequences [file 41420_2025_2666_MOESM1_ESM.docx]

**Primer sequences of genes for quantitative Real-Time PCR**

| Gene | Forward primer (5′-3′) | Reverse primer (5′-3′) |
| --- | --- | --- |
| *DCTPP1* | CGCCTCCATGCTGAGTTTG | CCAGGTTCCCCATCGGTTTTC |
| *CCND1* | GCTGCGAAGTGGAAACCATC | CCTCCTTCTGCACACATTTGAA |
| *CCNE1* | AGAGGAAGGCAAACGTGACC | TATTGTCCCAAGGCTGGCTC |
| *TP53I3* | CTGAACCGGGCGGACTTAAT | CCCAAAATGTTGCTGGCTCC |
| *AOX1* | GGGGTGTTCCGTGTTTTTCG | TCCGGGGTCAGTGGACTATT |
| *GADD45A* | AGCAGAAGACCGAAAGCGAC | TTGATGTCGTTCTCGCAGCA |
| *MAPK9* | GAAACTAAGCCGTCCTTTTCAGA | TCCAGCTCCATGTGAATAACCT |
| *CASP7* | AGTGACAGGTATGGGCGTTC | CGGCATTTGTATGGTCCTCTT |
| *BBC3* | AATTTGGCATGGGGTCTGCC | CCACAAATCTGGCAGGGGAC |
| *BCL2L1* | GGGCATTCAGTGACCTGACA | TCCACAAAAGTATCCTGTTCAAAGC |
| *BCL6* | CATCTCGGCTCAATTTGCGG | CCGGCTGTTGAGGAACTCTT |
| *AUF1* | GCGTGGGTTCTGCTTTATTACC | TTGCTGATATTGTTCCTTCGACA |
| *CDKN1A* | ACATCGCCAAGGAAAAACGC | GTCTGTTTCGGTACTGTCATCC |
| *MYC* | CACCTTGTAGCACGTCCTG | GACTCCCCAAGATGTGGTGG |
| *IL6* | ACTCACCTCTTCAGAACGAATTG | CCATCTTTGGAAGGTTCAGGTTG |
| *ACTIN* | GGACTTCGAGCAAGAGATGG | AGCACTGTGTTGGCGTACAG |
