# Supplementary figures and images for: DCTPP1 regulates oxidative stress homeostasis via AUF1 in human villous trophoblasts
Source: Cell Death Discov. 2025 Aug 23;11:400. doi: 10.1038/s41420-025-02666-8 (PMC12375105; doi:10.1038/s41420-025-02666-8)

Fig.1B


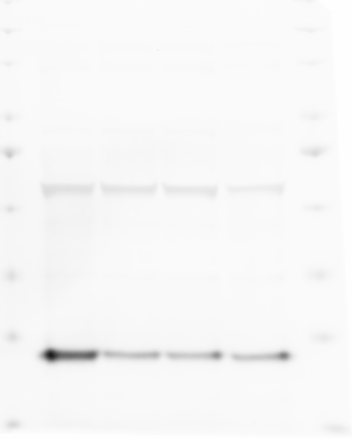
DCTPP1


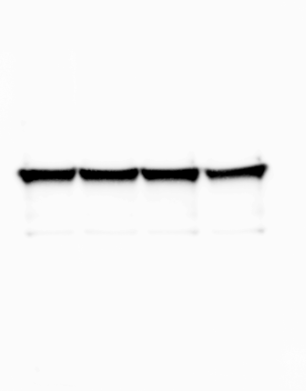
GAPDH

Fig.3H


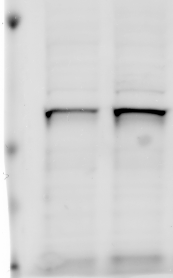
TP53I3


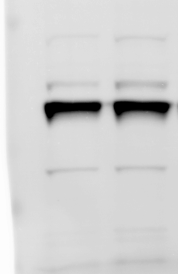
GAPDH

Fig.4B


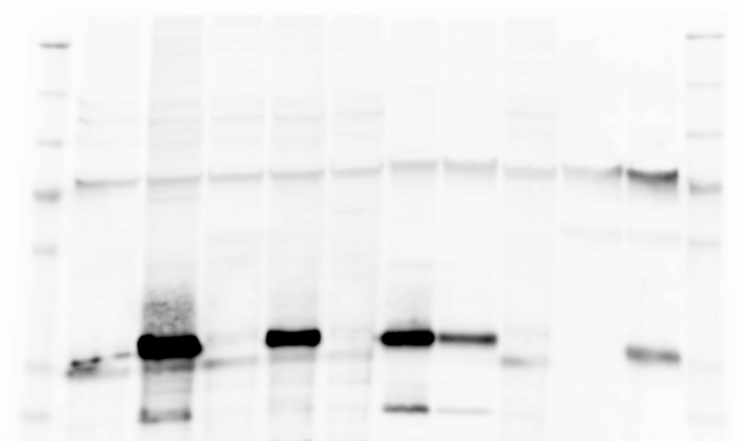
HA


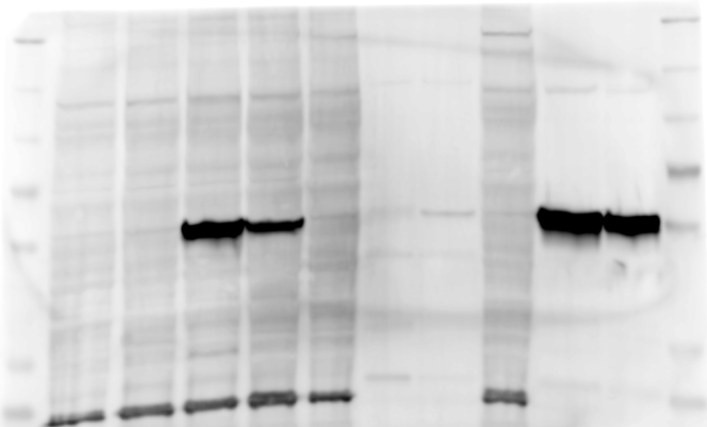
Flag

Fig.5A


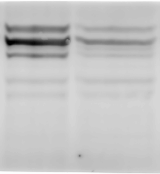
AUF1


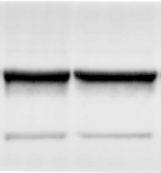
GAPDH

Fig.6H


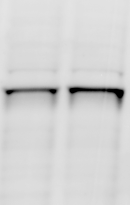
TP53I3


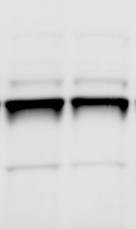
GAPDH

Supplement: Supplementary file 2 — Original western blots [file 41420_2025_2666_MOESM2_ESM.docx]
